# Fast or Slow? Compressions (or Not) in Number-to-Line Mappings

**DOI:** 10.1371/journal.pone.0120423

**Published:** 2015-03-27

**Authors:** Victor Candia, Paola Deprez, Jannis Wernery, Rafael Núñez

**Affiliations:** 1 Collegium Helveticum, Zurich, Switzerland; 2 Zurich University of the Arts, FSP Musikalische Interpretation, Zurich, Switzerland; 3 Department of Cognitive Science, University of California San Diego, San Diego, California, United States of America; Birkbeck, University of London, UNITED KINGDOM

## Abstract

We investigated, in a university student population, spontaneous (non-speeded) fast and slow number-to-line mapping responses using non-symbolic (dots) and symbolic (words) stimuli. Seeking for less conventionalized responses, we used anchors 0–130, rather than the standard 0–100. Slow responses to both types of stimuli only produced linear mappings with no evidence of non-linear compression. In contrast, fast responses revealed distinct patterns of non-linear compression for dots and words. A predicted logarithmic compression was observed in fast responses to dots in the 0–130 range, but not in the reduced 0–100 range, indicating compression in proximity of the upper anchor 130, not the standard 100. Moreover, fast responses to words revealed an unexpected significant negative compression in the reduced 0–100 range, but not in the 0–130 range, indicating compression in proximity to the lower anchor 0. Results show that fast responses help revealing the fundamentally distinct nature of symbolic and non-symbolic quantity representation. Whole number words, being intrinsically mediated by cultural phenomena such as language and education, emphasize the invariance of magnitude between them—essential for linear mappings, and therefore, unlike non-symbolic (psychophysical) stimuli, yield spatial mappings that don’t seem to be influenced by the Weber-Fechner law of psychophysics. However, high levels of education (when combined with an absence of standard upper anchors) may lead fast responses to overestimate magnitude invariance on the lower end of word numerals.

## Introduction

Numerical estimation research has shown that, among people in the industrialized world, number-to-space mappings are highly intuitive. Although this intuition is not universal [1], in the West even kindergarteners when asked to locate numbers on a line marked with a 0 on the left and 100 on the right, are reported to promptly place smaller numbers at left of the segment and greater numbers at right [2, 3]. The mapping, however, is not linear, as children allocate more space to small numbers and less to big numbers in a non-linear compressed manner. This non-linear compression is even more pronounced in children with mathematical learning disabilities [4, 5]. The data, in part, support the idea that numerical estimation follows the domain-general psychophysical Weber-Fechner law that subjective sensation increases proportional to the logarithm of the stimulus intensity (but see [6, 7] for alternative interpretations). It has been documented that with education and mathematical training, the development of mapping patterns starts to shift gradually, between kindergarten and fourth grade, from a logarithmic pattern to a primarily linear one [2, 3] (but see [8] for a different interpretation, where children’s responses are better modeled by two separate linear representations, one for units and one for tens). Besides, studies analyzing data with educated western participants have shown that when reporting on a line, participants exhibit a linear pattern in two main situations: [1] when the stimuli are symbolic (e.g., words)—which are intrinsically cultural, and [2] when they are non-symbolic (primarily psychophysical) as long as they are presented visually, and are easy to evaluate (e.g., small numerosity of dots in the 1–10 range). As it is argued elsewhere [9], the latter case, in fact, reproduces the conditions in which the learning of the number line occurs, that is, via the support of *visible*, *discrete* entities (e.g., dots or jumps performed by a bunny), presented in *small quantities* [9].

By contrast, the mappings have logarithmic compression when stimuli are non-symbolic and are hard to evaluate (e.g., large numerosities of dots, or tones presented acoustically), which by virtue of being primarily psychophysical in nature make them more susceptible to be influenced by the Weber-Fechner law. In line with these results a study has shown that even in the range of small numbers, attention is necessary for producing linear mappings onto the line: when attentional resources are taxed, the resulting mappings are logarithmic, even in educated participants [9]. Taken together, these studies suggest that the main factor responsible for the linearization of the number-to-space mappings is, ultimately, a learned cultural practice. A specific practice that emphasizes the distance invariance between whole numbers and prescribes the use of a line segment as a notational device for spatially characterizing such magnitude invariance. Indeed, *educated* participants (who have been systematically trained in learned cultural practices) by default exhibit linear mappings, but it is only when their attentional resources get taxed—and therefore their culturally learned practices get less monitored—that they begin to exhibit logarithmic compression [10]. This interpretation is further supported by research that has shown that when reporting *non-spatially*—i.e., via non-culturally developed forms such as squeezing a dynamometer, hitting a bell, or vocalizing at various intensities—university students in the West consistently produce (1) logarithmic mappings for *all* non-symbolic stimuli, including small number of dots (which when reported on a line it yields linear mappings), but (2) linear mappings for symbolic stimuli (words) in all tested non-spatial reporting forms [10].

One possibility for investigating the parameters that influence the forms that take number and numerosity mappings in general, and number-to-space in particular, is to assess participants’ responses at different levels of automatism. For instance, having fast responses from participants may indicate more *native* [9], instinctive, less monitored responses than slow responses, which may benefit from meta-cognitive processes as well as calculation and attentional resources [9]. But, surprisingly, with rare exceptions [10, 11] most of the literature on numerical estimation research and number-line mappings reports results in which the time given to participants for responding has not been explicitly considered (see for instance [1–3, 9, 12–15]). One of the main goals of the present study is to investigate in an educated western population, the role played by response time in shaping the number-to-space mappings. We hypothesized that, if linearization of number-to-line mapping responses is primarily a product of cultural practices such as education and training, then non-linear mappings, especially with non-symbolic stimuli, should be primarily observed in the more instinctive fast responses, which, being less monitored are relatively more exposed to the Weber-Fechner law of perception.

Another factor that seems to influence the shape of number and numerosity mappings is the magnitude of [6], and familiarity with number stimuli and anchors on the line [6, 7], especially the upper anchor [12]. Besides, non-linear compression patterns don’t seem to follow specific canonical magnitudes, but rather they appear to depend on proportional distances between anchors [6, 16]. Thus, reported logarithmical patterns exhibiting compression towards the upper end of a 0–100 scale may not correspond to a canonically compressed mental representation of the numbers in the vicinity of 100 proper, but rather depend on the magnitude of the upper anchor, which in this case would happen to be 100. In this study we want to further investigate this phenomenon, by using a less familiar upper anchor greater than the standard 100, namely, 130. We predicted that if the compression really depends on the upper anchor (i.e., the magnitude distance between the anchors), then when participants provide responses over the entire 0–130 range the compression of fast response mappings should be detected over the whole 0–130 range, but not within the 0–100 range.

To avoid artificially speeded responses we investigated spontaneous responses where ‘fast’ and ‘slow’ responses were categorized following relevant reaction time criteria from the literature in number cognition (e.g., [17]). In order to systematically investigate the role of the various variables playing a role in number-to-space mappings and the underlying mental representations, in this study we used both, non-symbolic (dots) and symbolic (words) stimuli. We used words rather than Arabic numerals for symbolic stimuli to match the minimal arithmetical affordances of the non-symbolic stimuli (dots): words, unlike Arabic numerals, are not readily available for arithmetical calculations and digit-based algorithms, and therefore reduce the potential of invoking calculation-related confounds [10].

## Material and Methods

### Ethics Statement

This study was approved by the ethics committee of the ETH Zurich. All participants gave their written informed consent and were treated according to the Helsinki convention for the treatment of experimental subjects (http://www.wma.net). Participants received monetary reward for their participation.

### Participants

Fifty-four students from two large higher education institutions in Zurich (ETH Zurich and University of Zurich), Switzerland, (range 19–40), 26 women (mean age = 24.1, SD = 4.6) and 28 men (mean age = 24.2, SD = 4.3), participated in the study. All participants were self-reportedly right-handed and their native language was German. They had normal or corrected-to-normal vision, had no disorders or disturbances of the central nervous system (including psychiatric disorders), no disorders of the arm, hand and or fingers, and were not under the influence of any medication.

### Stimuli and materials

For data collection and stimulus presentation, a custom-made software was implemented using Psychtoolbox 3 for MatLab7. Explanations and stimuli were displayed on a computer screen (BenQ GL2450HM) which had a computer mouse connected to its computer (Logitech M-UAS144 LS1 Laser Mouse). For the target task, a black horizontal line was depicted (440 pixel length, 5 pixel thickness) over a white background, with numerical anchors at each side above the line, 0 on the left and 130 on the right (using corresponding German words for the symbolic stimuli presentation, and using an empty circle and a circle (radius 90 pixel) with 130 dots, respectively, for the non-symbolic stimuli presentation). Dots had a constant size (radius 5 pixel) and dot patterns were drawn on-line on random positions within the circle. Response time (RT)—i.e. the time between the presentation of a number stimulus and the corresponding response on the line—was recorded automatically. Stimuli were number stimuli between 10 and 130 (inclusive) in steps of 10, presented either symbolically as written German words (Swiss German spelling), or non-symbolically as collections of dots contained within a circle. Number of letters in words varied from 4 (German word for 10) to 15 (German word for 130) [1 number had 4 letters, 7 numbers had 7 letters, 1 number had 8 letters, 1 number had 11 letters, 1 number had 14 letters, 1 number had 15 letters; Mean 8.3 letters, SD 3.1].

### Procedure

Participants were assessed individually, while sitting in an acoustically shielded room in front of a computer screen placed 70 cm away at eye-level. Instructions for the participants were displayed on the computer screen and, if needed, verbally clarified by the experimenter. Participants were asked to point via a mouse click to the location on the horizontal line corresponding to each of the number stimuli they were presented on the screen, and they were simply instructed to concentrate and answer ‘quickly’. This was done in order to later analyze spontaneous ‘fast’ and ‘slow’ responses not affected by stress associated with artificially forced or speeded responses. The line and the stimuli were presented simultaneously. After each response, line and number stimuli disappeared right after the participant clicked on the line. To dilute memory effects from one number stimulus presentation to the next, there was, after each response, a short break of 4s displaying a blank screen. We used one experimental block per stimulus condition, each consisting of 3 randomized presentations of each number stimulus (3x13 stimulus presentations in total for each condition). Number of repetitions of stimulus presentations was kept low to avoid lengthening experimental times, which entail the risk of fatiguing the participants. Runs of non-symbolic and symbolic stimuli were counterbalanced across subjects and number stimuli were randomized. Previous to the main experimental session, to familiarize participants with the tasks, there was a short training with five stimuli not included in the experimental set. The total duration of the experiment was ca. 20 min per participant (comparable to other studies, e.g., [17, 18]).

### Data preparation

To verify the validity of response location for a given type of stimulus, for each participant we calculated multiple regressions with linear and logarithmic predictors on mean response location (each based on three responses for every number stimulus on the entire range 10–130). We excluded two participants from the analysis of responses to non-symbolic stimuli whose responses showed neither a linear nor a logarithmic pattern (i.e., the weight of neither *B*_lin_ nor *B*_log_ coefficients was statistically significant). To verify the validity of response range—i.e., the use of a sufficiently long extension of the line to locate responses—we calculated, for each participant, the response range based on median responses. We excluded one participant from the analysis of responses to symbolic stimuli whose responses used less than 75% of the extension of the line. To make sure that participants kept stable levels of engagement and motivation throughout the experiment, we performed a split-half reliability test for each stimulus condition, comparing the mean over median RTs of the ensemble of trials in the first half of the experimental blocks with the corresponding mean in the second half. No differences in RTs were found (dots: *t*(52) = -0.944, *p* = 0.349; words: *t*(52) = 1.779, *p* = 0.081).

Spontaneous ‘fast’ and ‘slow’ responses were defined with respect to relevant criteria taken from number cognition studies involving reaction time (e.g., [6, 17, 19, 20]). For non-symbolic stimuli (dots), a response was considered to be fast if its reaction time (RT) was RT≤ 2000 ms and it was considered to be slow if 2000 ms < RT < 3500 ms. For symbolic stimuli (words), a response was considered to be fast if RT ≤ 2500 ms and it was considered to be slow if 2500 ms < RT < 4000 ms. Slightly larger RTs cut-offs (+500 ms) were used for symbolic stimuli in order to account for the fact that words require reading (and in German they contain many characters), implying thus longer processing times.

The criteria underlying these cut-offs were the following. First, a time window of ca. 1400 ms has been used in simple tasks such as in SNARC-related studies (e.g., 1300 ms [17], 1500 ms [19], 1350 ms [20]), where (1) participants simply respond via directly pressing one of two buttons positioned immediately next to corresponding fingers, and (2) where speeded responses are emphasized [17]. Second, in some number-line reports (e.g., [6]), where participants must carefully point (with a finger or cursor manipulated with a computer mouse) on a line segment, 500 ms has been considered to be the minimum for a response to be valid. Given that in the present study extra time was needed to move a computer mouse and that participants were spontaneously providing un-speeded responses, we considered that a cut-off of 2000 ms for non-symbolic stimuli appropriately divided slow from fast spontaneous responses. This *de facto* left a 1500 ms time window for acceptable fast and slow, responses. Regarding non-symbolic stimuli, we used slightly larger RT cut-offs (+500 ms) in order to account for a number of factors. First, word processing requires longer times. Verbal numerals, for instance, have been shown to go through additional transcoding operations as compared to Arabic numerals [17]. Second, it as been discussed that the recognition of printed words require identifying symbols from other orthographic competitors, linking the symbols to semantics, which need to be integrated to conceptual content [19]. And third, words with a length of 8 letters or more (as it is the case in the present report) are always fixated—sometimes more than once—and natural reading fixations, which occur between eye saccades, have a range of 200–300 ms with a higher range of variability up to 500 ms [20] and a standard deviation of 70 ms [19].

For a participant to be classified, as having either fast or slow responses for a given stimulus type, and to be eligible for further analysis, s/he had to (1) have at least 10 out of 13 possible number stimuli with data satisfying the time criterion stated above, and (2) have at least a statistically significant weight of either *B*_lin_ or *B*_log_ coefficients in the individual multiple regression with linear and logarithmic regressors on the median time-categorized responses. As a result of this procedure, four groups were constituted: A set of responses coming from participants who spontaneously responded fast to non-symbolic stimuli (n = 11), to symbolic ones (n = 13), who spontaneously responded slow to non-symbolic stimuli (n = 34), and to symbolic stimuli (n = 18).

## Results

**Fig 1:**
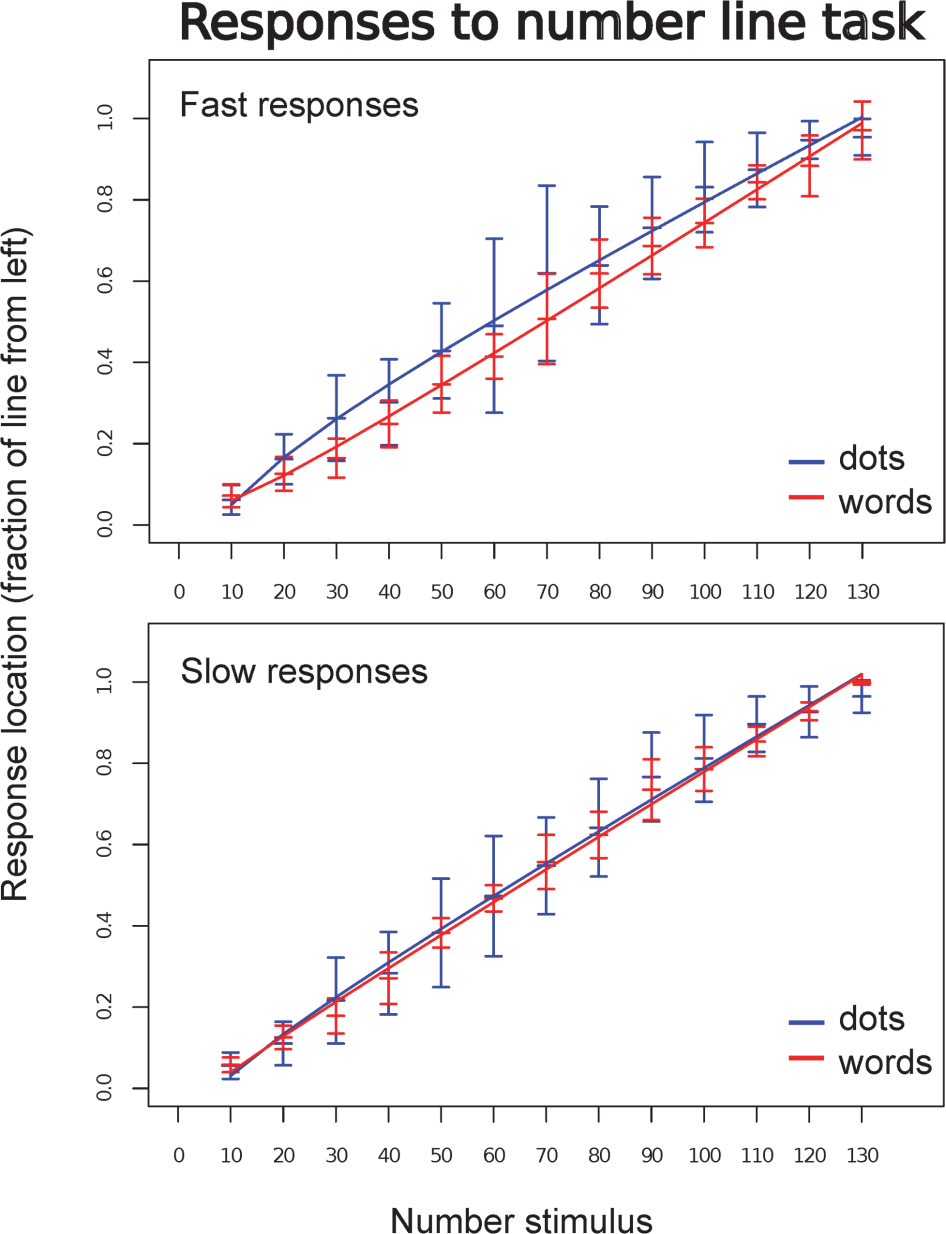
Responses to the number line task. Mean response locations with standard error of the mean are shown, separated by fast (top) and slow (bottom) responses, as well as by type of stimuli—non-symbolic (dots) in blue and symbolic (red) in red. The fitted regression lines for each case are taken from a multiple regression analysis of response location that included linear and logarithmic regressors. Non-linear compression—positive for dots and negative for words—is only observed in fast responses (see details in text, and in Fig. 2).

The mean response locations provided by the participants over the 10–130 range were analyzed by means of ordinary least squares (OLS) multiple regressions with linear and logarithmic predictors (Fig. 1).

Slow responses, for both, non-symbolic (dots) and symbolic (words) stimuli, exhibited a linear pattern (dots: *B*_lin_ = 0.007, st. error = 0.001, *t* ratio = 10.344, *df* = 10, *p* < 0.001; words: *B*_lin_ = 0.008, st. error < 0.001, *t* ratio = 16.516, *df* = 10, *p* < 0.001) and showed no evidence of a non-linear compression over and above the linear one (dots: *B*_log_ = 0.098, st. error = 0.083, *t* ratio = 1.181, *df* = 10, *p* = 0.265; words: *B*_log_ = 0.034, st. error = 0.056, *t* ratio = 0.620, *df* = 10, *p* = 0.549). Fast responses, however, exhibited a different pattern. While responses to symbolic stimuli (words) showed a linear pattern (*B*_lin_ = 0.008, st. error < 0.001, *t* ratio = 17.875, *df* = 10, *p* < 0.001) with no evidence of non-linear compression (*B*_log_ = -0.076, st. error = 0.055, *t* ratio = -1.378, *df* = 10, *p* = 0.2), responses to non-symbolic stimuli (dots) did exhibit a non-linear compression over and above the linear one (*B*_lin_ = 0.006, st. error = 0.001, *t* ratio = 9.78, *df* = 10, *p* < 0.001; *B*_log_ = 0.179, st. error = 0.075, *t* ratio = 2.378, *df* = 10, *p* = 0.039).

The difference in nonlinear compression between responses to non-symbolic and symbolic stimuli was further corroborated with OLS multiple regressions containing linear and logarithmic regressors and an added “dummy variable” (*D*)—a technique widely used in other domains [21]—aimed at evaluating the discriminability between responses to non-symbolic and symbolic stimuli with respect to logarithmic compression. When considering the ensemble of fast responses (symbolic and non-symbolic responses combined: 2 stimulus type x 13 number stimuli = 26 observations) the weight of the linear regressor was significant (*B*_lin_ = 0.007, st. error = 0.001, *t* ratio = 11.890, *df* = 23, *p* < 0.001), indicating shared linear structure, with no evidence of shared logarithmically compressed structure (*B*_log_ = 0.052, st. error = 0.073, *t* ratio = 0.711, *df* = 23, *p* = 0.484). Crucially, however, when adding the dummy variable (with *D* = 1 if the observation was a response to a non-symbolic stimulus, and *D* = 0 if it was a response to a symbolic stimulus) the weight of the dummy x logarithmic interaction was highly significant (*B*_*D* x log_ = 0.029, st. error = 0.006, *t* ratio = 4.727, *df* = 22, *p* < 0.001), confirming that significant differences in logarithmic compression do exist between fast responses to non-symbolic and symbolic stimuli. Slow responses, on the contrary, did not exhibit such differences in nonlinear compression. Slow responses expectedly shared linear structure (*B*_lin_ = 0.008, st. error < 0.001, *t* ratio = 18.578, *df* = 23, *p* < 0.001) with no evidence of non-linear compression (*B*_log_ = 0.066, st. error = 0.048, *t* ratio = 1.385, *df* = 23, *p* = 0.179). But, importantly, the weight of the dummy x logarithmic interaction was not significant (*B*_*D* x log_ = 0.005, st. error = 0.006, *t* ratio = 0.88, *df* = 22, *p* = 0.388), indicating no evidence that there is a significant difference in logarithmic compression between fast responses to non-symbolic and symbolic stimuli.

**Fig 2:**
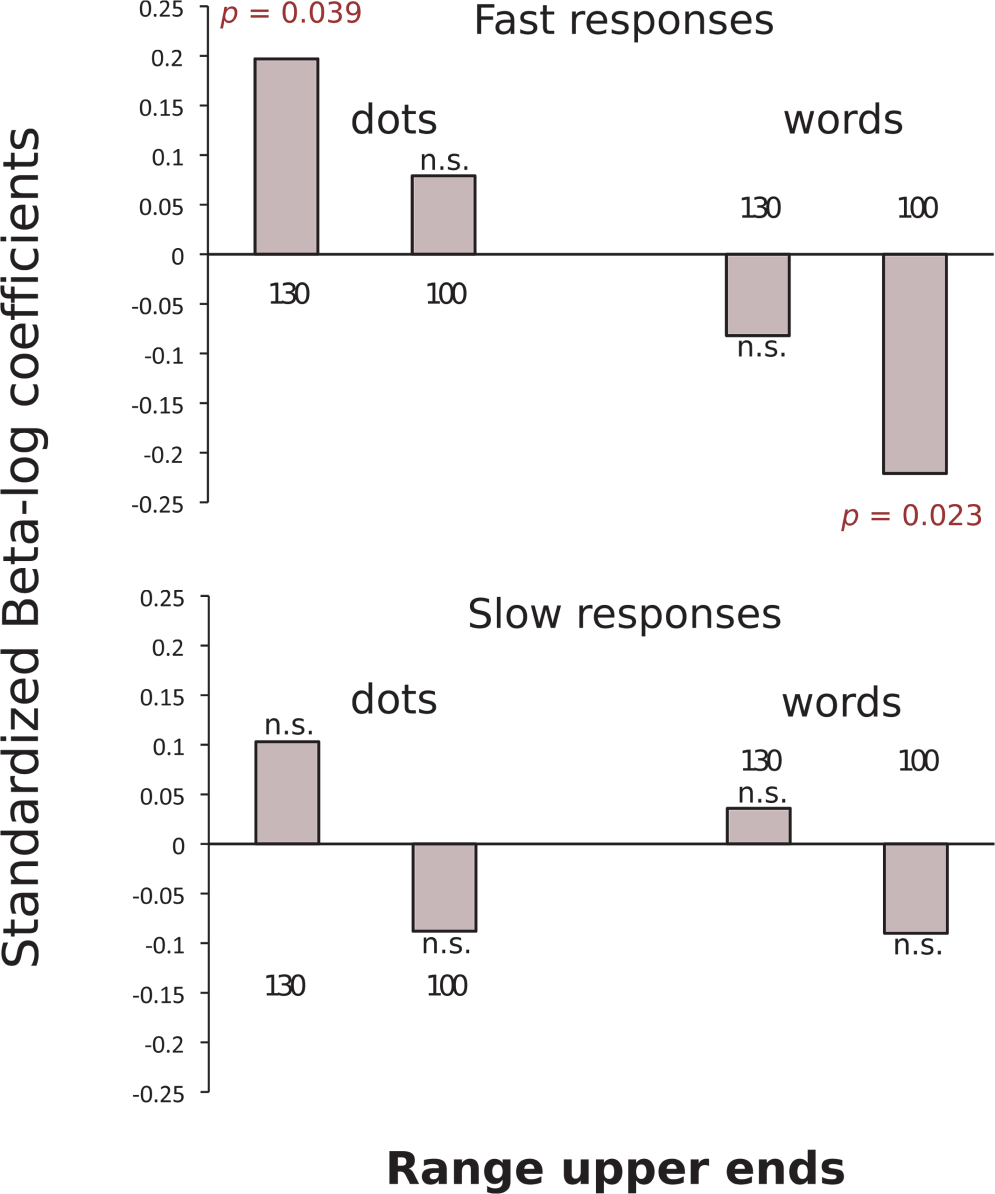
Standardized Beta-log coefficients for fast and slow responses. The values are taken from a multiple regression analysis of response location that included linear and logarithmic regressors. Responses to non-symbolic stimuli (dots) appear on the left, separated by the range under consideration, 0–130 or 0–100, labeled by their upper bound ‘130’ and ‘100’ respectively. Responses to symbolic stimuli (words) appear on the right. Slow responses only produced linear mappings with no evidence of non-linear compression. Fast responses revealed distinct patterns of non-linear compression for non-symbolic and symbolic stimuli. A significant non-linear (logarithmic) compression was observed in responses to non-symbolic stimuli in the entire range 0–130, but not in the reduced 0–100 range, whereas a significant negative compression was observed in responses to symbolic stimuli in the reduced range 0–100, but not in the entire 0–130 range.

In order to analyze the significance of the compression in the range 10–100 (for the responses which had been produced over the whole line segment anchored with 0 and 130), we ran further multiple regressions with linear and logarithmic regressors for this restricted lower range. As in the case of slow responses over the entire 0–130 range, slow responses in the 0–100 range exhibited only a linear pattern for both type of stimuli (dots: *B*_lin_ = 0.009, st. error = 0.001, *t* ratio = 1.082, *df* = 7, *p* < 0.001; words: *B*_lin_ = 0.009, st. error = 0.001, *t* ratio = 14.962, *df* = 7, *p* < 0.001) with no evidence of non-linear compression (dots: *B*_log_ = -0.073, st. error = 0.052, *t* ratio = -1.406, *df* = 7, *p* = 0.202; words: *B*_log_ = -0.072, st. error = 0.058, *t* ratio = -1.237, *df* = 7, *p* = 0.256). Fast responses over the 0–100 range did not provide evidence of a significant non-linear compression when obtained with non-symbolic (dots) stimuli (*B*_lin_ = 0.008, st. error = 0.001, *t* ratio = 9.44, *df* = 7, *p* < 0.001; *B*_log_ = 0.063, st. error = 0.078, *t* ratio = 0.811, *df* = 7, *p* = 0.444). However, when obtained with symbolic (words) stimuli they exhibited a significant negative non-linear compression (*B*_lin_ = 0.01, st. error = 0.001, *t* ratio = 15.867, *df* = 7, *p* < 0.001; *B*_log_ = -0.167, st. error = 0.058, *t* ratio = -2.907, *df* = 7, *p* = 0.023). Fig. 2 shows the standardized Beta-log coefficients of these analyses, for both, the 0–100 and 0–130 range.

## Discussion

The results of this study show that speed of response to number-to-space tasks reveal differences in mapping compression with respect to type of stimuli, and mapping range. Traditionally, response time has not been explicitly considered as an important parameter in number estimation and number line studies. The present report points to the importance of doing so, even when studying a university student population. Based on findings from previous studies [10, 13], we predicted that mappings of fast responses to dots (non-symbolic), but not to words (symbolic), would exhibit a logarithmic compression. The results confirm this prediction, and show that the difference in non-linear compression vanishes when responses are slow, since responses to both type of stimuli become linear. The data suggest that fast responses to dots, being more automatic and less monitored than slow responses, are more exposed to the Weber-Fechner law of perception, thus yielding logarithmic mappings.

Why do slow responses become linear? It has been suggested that with education and mathematical training, the development of mapping patterns starts to shift gradually, between kindergarten and fourth grade, from a logarithmic pattern to a primarily linear one [2, 3]. Linear mappings are observed in well-educated adults responding to symbolic (words) stimuli and to visual non-symbolic (dots) stimuli [10, 13]. But when responding to a large numerosity of dots [10, 13], or when affected by reduced attentional resources [9], the mappings re-appear as logarithmically compressed. This has led to the proposal that linear and logarithmic mappings might co-exist in an individual’s mind [9, 10, 13]—their manifestation depending on factors such as type of stimuli and availability of attentional resources—where the logarithmic ones are taken to be more instinctive or ‘native’ [9]. Our results show that spontaneous slow responses—especially when dots are concerned—seem to get the extra few hundred milliseconds needed to move away from the ‘native’ or instinctive state ruled by the Weber-Fechner law. This extra time allows for adjustments and meta-reflexions, which in the ontogeny of individuals is mediated and scaffolded via cultural factors such as language and education. This scaffolding ultimately results in linear mappings that match the ones observed with the culturally shaped symbolic stimuli: words. And why do words yield linear mappings, even when reporting non-spatially over a variety of media [10]? First, words for whole numbers bypass psychophysically-related phenomena such as attentional processes [9], and therefore are somewhat immune to logarithmically inducing pressures. And, most importantly, number words have the crucial property of encapsulating precise quantitative meaning that emphasizes *magnitude invariance*, between, for instance, the predecessor and the successor of any given counting number. In other words, meaning in number lexica has been culturally created to *have* a linear structure, property that is taught to individuals via language and education.

To minimize conventional responses that might mask compression differences due to response time and/or type of stimuli, our study used a 0–130 ‘number line’ rather than the standard 0–100. Based on the observation that non-linear compression patterns don’t seem to follow specific canonical magnitudes, but rather they depend on proportional distance between anchors [6, 16], and familiarity with the anchors and number stimuli [12, 14], we predicted that in case of a non-linear compression of fast response mappings, this should only be significant when evaluated over the whole 0–130 range, and not when evaluated over the restricted 0–100 range. In other words, we expected compression to be detectable at the higher end of the 0–130 range. Our results confirmed this prediction, as fast responses to dots produced a logarithmically compressed mapping detectable at the 0–130 range but not at the 0–100 range, which indicates a compression relative to, and in proximity of, the upper anchor 130, not the standard 100. The results give support to the proposal that non-linear compression in number-to-space mappings is not fixed with respect to specific standards (e.g., numbers in the vicinity of 100) but that it is shaped with respect to the number-line anchors. But our study also produced some unexpected results. Fast responses to words revealed a significant negative compression in the reduced range 0–100, but not in the 0–130 range, indicating compression in proximity to the lower anchor 0. Graphically (Fig. 1), the negative non-linear compression observed for symbolic stimuli (words) at the lower end of the line appears as a mirror opposite of the upper compression exhibited by responses to non-symbolic stimuli (dots). However, while dots are primarily psychophysical stimuli, susceptible to be influenced by the Weber-Fechner law, words are not. Dots and words are different in nature. We speculate that intensely educated university students, when perceiving symbolic stimuli and in the absence of a standard high anchor (which would have been 100) and conventionalized reference points [22, 23], might have been over-cautious in following the invariance magnitude principle. As a result, on the side of origin of the scale they might have over-compensated in leaving enough space for a more abundant number-locations than the standard 100 would have, which resulted in a significant compression at the lower end of the scale. In slow responses this compression disappeared as participants operated with more time, which allowed for adjustments leading to a more linear calibration. Further research is needed to test this explanatory proposal, but in doing so, it seems that investigating response time is a fruitful path to follow.

## Supporting Information

S1 Dataset Dots. (XLSX)

S2 Dataset Words. (XLSX)
